# Collected Papers by the Staff of St. Mary’s Hospital

**Published:** 1911-05

**Authors:** 


### BOOKS AND AUTHORS

**Collected Papers by the Staff of St. Mary's Hospital,** Mayo Clinic, Rochester, Minnesota. 1905-1909. Octavo of 688 pages. Illustrated. Philadelphia and London: W. B. Saunders Company. 1911. (Cloth, $5.50, net.)

This interesting book is a compliation of the various papers which have appeared from time to time from the staff of the St. Mary's hospital, and it provides a ready means of reference to the work of the various men connected with that famous institution. These papers have been printed in different medical journals, and the many unique methods and ideas which have been advocated have been accepted by the profession, in a very great measure, and have been incorporated by the surgeon in his work and technic with increasing satisfaction.

From the first article on "Epithelioma of the Lip," to the last one on "Bier's Congestive Therapy," the book contains papers on many different subjects, filled with points of great interest and of highly practical value. Judd recommends that in every epithelioma of the lip, whether it starts from a wart, ulcer, excoriation, tubercle or leukoplakia, the submental and submaxillary glands should always be removed, even if they are not involved so far as macroscopical examination can determine, and the method of operation is beautifully illustrated by satisfactory plates. He also recommends that the glands of the axilla be removed in every case of cancer, no matter how circumscribed the growth may be.

The article on diverticula of the esophagus with a report of six cases is very interesting and the results of operation are very encouraging. This is a rare affection, and to have such a group of cases is fortunate; the paper on cardiospasm with report of forty cases makes valuable reading.

The work of the Mayos on the stomach, duodenum, gall-bladder and pancreas, has been so universally accepted as scientific, logical and practical, that a perusal of these articles brings the subject to us, in a most conclusive and satisfactory manner.

The articles on hernia and practicularly the description and plates of the umbilical variety stamps their work as most ingenious, and shows a versatility in the knowledge of the laws of mechanics, which has made their radical cure operation so signally successful; and we believe that the operation is anatomically correct and surgically ideal. The Mayo incision for inguinal hernia possesses some advantages in subjects where the conjoined tendon is weak and atrophied, but we are satisfied that the cord should always be transplanted as recommended by Bassini and the Mayo statistics conclusively prove this contention, as they had four rěcurrences in four hundred (400) transplantation cases, and twenty-one in twelve hundred and forty-one (1241) cases, where the cord was left undisturbed.

The articles on the thyroid gland are very valuable, and scintillate with practical points. In fact, all of the papers in the book are based upon a study of such a vast number of cases and the deductions made from them, before and after operation, are so scientific that their conclusions are of inestimable value to every student of medicine and surgery, and we cheerfully recommend the book.

The volume is attractive in size, printed upon beautiful paper and the plates represent the highest type of the printer's art.

